# Retrosternal goiter masquerading as type II respiratory failure. A case report

**DOI:** 10.1016/j.ijscr.2022.107104

**Published:** 2022-04-20

**Authors:** Sohail Bakkar, Queen Hamdeh, Robert Haddadin, Gianluca Donatini, Theodosios S. Papavramidis, Mohamed Tawalbeh

**Affiliations:** a Department of General and Specialized Surgery, Faculty of Medicine, the Hashemite University, Zarqa 13133, Jordan; b Department of General and Endocrine Surgery, University of Poitiers, CHU Poitiers, Poitiers, France; c 1st Propaedeutic Surgical Department, University Hospital of Thessaloniki AHEPA, Aristotle University of Thessaloniki (AUTH), Thessaloniki 5462, Greece; d Department Surgery, Faculty of Medicine, Jordan University, Amman, Jordan

**Keywords:** Retrosternal, Intrathoracic, Mediastinal, Goiter

## Abstract

**Introduction and importance:**

Retrosternal Goiter (RG) represents a challenging clinical entity for surgeons. Although the vast majority of cases are successfully operated via a cervical access, there still remains a small minority that require an extra-cervical approach, even in experienced hands. Factors that shift the odds towards an extra-cervical approach are mainly related to the anatomic characteristics of the retrosternal mass.

**Case presentation:**

We herein report a case of RG presenting as type 2 respiratory failure without a palpable neck mass, in an 81-year-old female. Despite her history of subtotal thyroidectomy for Graves' disease, the patient's chest x-ray showed a central mediastinal mass shifting the trachea to the left. The retrosternal mass extended below the aortic arch, and carina on computed tomography. It also extended into the posterior mediastinum. All these anatomical features of the RG along with the patient's previous neck surgery were in favor of an extra-cervical approach. Nevertheless, a cervical approach was attempted, and was concluded successfully.

**Conclusion:**

CT plays a key role in determining the likelihood of requiring an extra-cervical approach in RG. Even if the odds seem to be in favor of an extra-cervical approach, an attempt to remove the goiter through a cervical incision should always be made by an experienced surgeon, using all available techniques, and taking all required precautions, on account of less risk of surgical and aesthetic damage obtained with this approach.

## Highlights

• Respiratory failure is an unprecedented manifestation of retrosternal goiter (RG).
• CT plays an important role in determining the likelihood of requiring an extra-cervical approach for RG.
• An ectopic goiter, previous neck surgery, extension below the level of the carina and/or into the posterior mediastinum favor an extra-cervical approach.
• A cautious attempt to remove RG through a cervicotomy should always be made, on account of reduced risk of surgical and aesthetic harm with this approach.
• If RG is performed by a skilled surgeon, familiar with its unique pitfalls, the assistance of a thoracic surgeon may seldom be required.

## 1 Introduction

Retrosternal Goiter (RG) continues to pose a challenge for surgeons. Although the vast majority of cases are successfully operated via a cervical access, there still remains a small minority that require an extra-cervical approach, even in experienced hands. Factors that shift the odds towards an extra-cervical approach are mainly related to the anatomic characteristics of the retrosternal mass. Nevertheless, even if the odds are strongly in favor of an extra-cervical approach, a cautious attempt to remove RG through a cervicotomy should always be made, on account of reduced risk of surgical and aesthetic harm.

This case was managed in an academic tertiary referral center. It demonstrates a stepwise approach in evaluating retrosternal goiter in terms of surgical strategy. It also demonstrates the importance of cautiously attempting a cervical approach against all odds to achieve the patient's best interest. This work has been reported in line with the SCARE 2020 criteria [1].

## 2 Case presentation

**Fig. 1:**
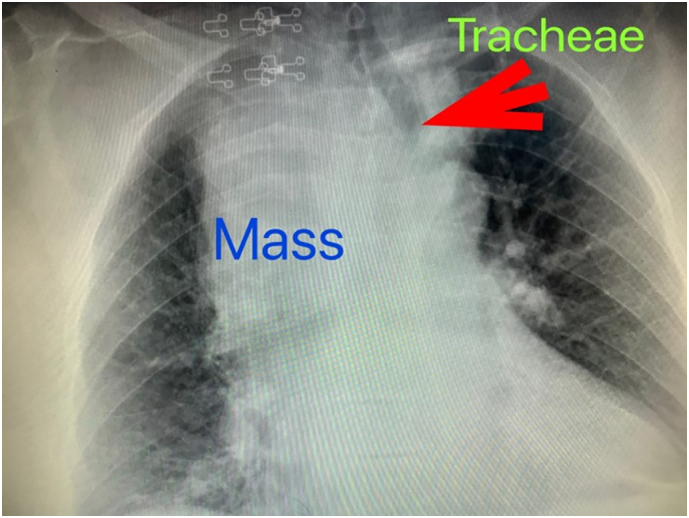
Chest x-ray demonstrating a widened mediastinum with tracheal deviation to the left.

**Fig. 2:**
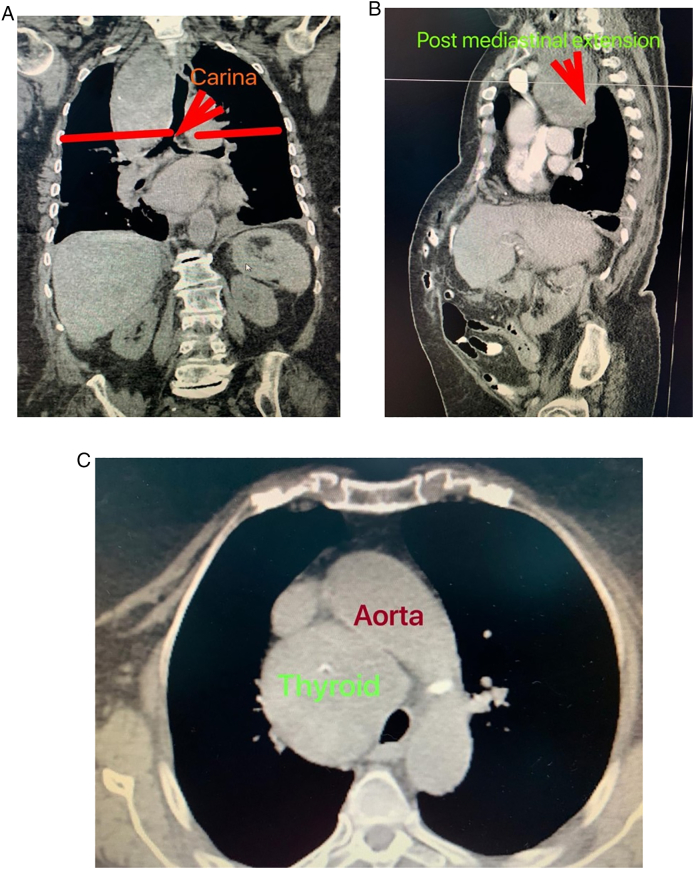
(A) Coronal view: extension below the carina (B) Sagittal view: extension into the posterior mediastinum (C) Axial view: extension below the aortic arch.

**Fig. 3:**
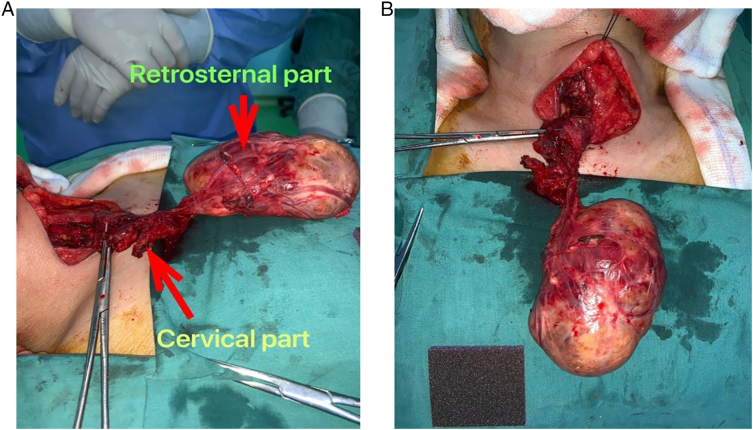
(A) Lateral view of the resected right thyroid lobe with its cervical and retrosternal components. (B) Superior view of the surgical specimen.

**Fig. 4:**
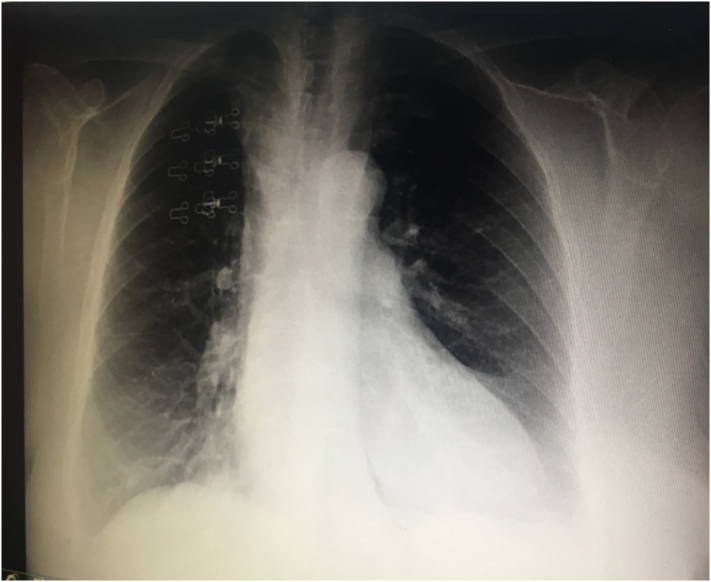
Post-operative chest x-ray with a centrally located trachea and no mediastinal mass.

**Fig. 5:**
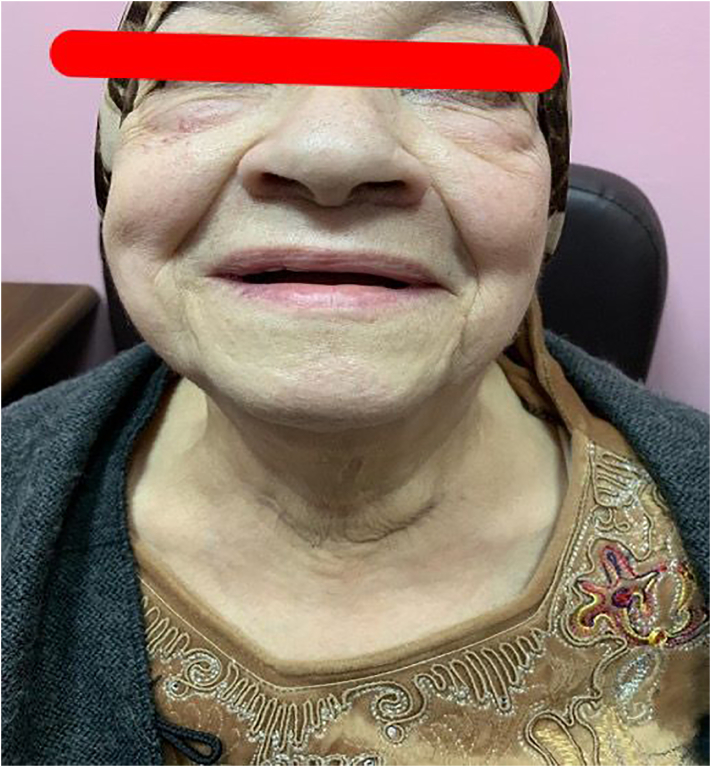
The patient one-week post-surgery with a cosmetically favorable result. This image is published with the patient's consent.

We herein report the case of an 81-year-old hypertensive female who was admitted to the Intensive Care Unit (ICU) at our institute (an academic tertiary referral center), in January 2019, and managed by the pulmonary and critical care team as a case of type II respiratory failure. On admission her oxygen saturation was 78% on rom air, and her arterial blood gases (ABGs) were: pH = 7.32, PaCO_2_ = 61 mmHg, Pa O_2_ = 45 mmHg, and HCO3 = 33 mEq/L. The ABGs readings reflected acute on top of chronic hypercapnic respiratory failure. Surgical consultation was received upon the discovery, on chest x-ray (CXR), of a large mediastinal mass narrowing the trachea and displacing it to the left (Fig. 1). The patient reported a history of subtotal thyroidectomy for Graves' disease. However, she was not on thyroxine replacement therapy and her thyroid stimulating hormone (TSH) level was 0.7 IU/L (normal range 0.35–3.0 IU/L). Her main complaint was shortness of breath of 4-month duration, but, no dysphagia, or dysphonia. She had no palpable mass in her neck. The radiographic appearance was classical for retrosternal goiter (RG). However, in view of her history of thyroid surgery we opt to confirm the thyroid origin of the mass by scintigraphy prior to proceeding to computed tomography (CT) with intravenous contrast to avoid thyroid stunning (Wolf-Chaikoff effect) [2]. Indeed, thyroid origin was confirmed by scintigraphy. CT of the neck and chest, with the arms on the sides, was then performed for further characterization of the retrosternal extension, and to assess the need for sternotomy. It demonstrated a secondary right-sided RG extending below the carina, as well as into the posterior mediastinum. The volume of the RG was estimated to be 375 mL on CT (Fig. 2). Despite the history of a previous neck surgery and the considerable anatomical extent of the mass in the mediastinum, the decision was made to operate via a cervicotomy. Nevertheless, on-site Thoracic Surgeon standby was arranged. Prior to surgery, the pulmonary team commenced non-invasive ventilation by BI-PAP for 5 days. The patients' ABGs improved to become: pH = 7.42, PaCO_2_ = 50 mmHg, Pa O_2_ = 57 mmHg, and HCO3 = 38 mEq/L, and her oxygen saturation became 89% on room air. *trans*-Nasal flexible fiberoptic laryngoscopic evaluation of the vocal folds demonstrated normal and symmetrically mobile vocal cords. The American Society of Anesthesiology (ASA) category 3 patient underwent fiberoptic intubation followed by a successful right-sided hemithyroidectomy conducted through the neck using the LigaSure™ small-jaw sealer and divider. The procedure was performed by a high-volume Endocrine Surgeon (SB) who has an annual case-load in excess of 300 procedures. The procedural steps of the operative technique applied included: 1. Section of the superior pedicle 2. Division of the isthmus for further mobilization and medialization of the thyroid lobe 3. Identification of the recurrent laryngeal nerve (RLN) superiorly, and dissecting it caudally 4. Release of lateral thyroid attachments using the energy-based device 5. Concomitant pulling of the gland using the left hand, and blunt dissection of lateral and postero-lateral adhesions using the right hand which aids in delivering the gland from the thorax. It is worth mentioning at this point that the distal edge of the RG was way beyond the reach of the surgeon's finger. This implies a cautious and meticulous dissection and delivery of the lobe due to possible attachments to great vessels in the thorax beyond the surgeon's visual field and reach. Therefore, forceful delivery in the presence of resistance should always be avoided not to inflict harm resulting from potential inadvertent intrathoracic vascular injury. A size 10 Readivac® drain was placed. Operative time was 30 min from incision to closure. Fig. 3 demonstrates the resected right thyroid lobe. The patient's respiratory parameters, on the anesthesia monitor, improved immediately following resection of the RG. The patient was transferred to the ward. Post-operatively, her oxygen saturation on room air was 96%, and her ABGs were: pH = 7.42, PaCO_2_ = 42 mmHg, Pa O_2_ = 77 mmHg. Her post-operative chest x-ray is displayed in Fig. 4. The drain-output was 30 cc of serosanguinous fluid on the morning following surgery, therefore, it was removed. She was discharged on the first post-operative day without morbidity. Thyroxine replacement therapy was given applying the weight adjusted dose for elderly (1.2 x Body weight). One-week post-surgery the patient expressed complete satisfaction with the outcome of her surgery, and was content with the cosmetic appearance of her surgical site (Fig. 5). The histologic examination of the surgically resected specimen reported that the specimen weighed 497 g, and that the longitudinal axis of the retrosternal component measured 15 cm. There was no evidence of malignancy.

## 3 Discussion

“The extirpation of the thyroid gland for goiter typifies perhaps better than any other operation the supreme triumph of the surgeon's art.”

William Halsted, 1852–1922 [3]

A century of dedication and hard work was required to transform a procedure that was associated with considerable morbidity and mortality into one labeled as “extremely safe and efficient”. Indeed, Kocher's thyroidectomy symbolizes a triumph for surgery as a blend of art and science [3]. Still, RG that is documented in up to 20% of thyroidectomies, represents a challenging clinical entity for surgeons. There is no uniform definition for RG. However, the most commonly accepted one is that of a goiter with ≥50% of its mass being located in the mediastinum [4]. This clinical entity is broadly classified into primary and secondary RG. The former represents ectopically located thyroid tissue in the mediastinum (i.e. its presence in the mediastinum is congenital). Therefore, it is not connected to the thyroid gland in the neck, and receives its blood supply from mediastinal vessels. Primary RG accounts for less than 1% of RGs. On the other hand, secondary RG represents downward migration of thyroid tissue originating from the cervical part of the gland into the mediastinum. This migration is facilitated by gravity, the negative intra-thoracic pressure, and anatomical barriers (thyroid cartilage, strap muscles, and vertebral bodies) that prevent enlargement in other directions [5], [6], [7].

The clinical manifestations of RG range from an asymptomatic neck mass to considerable compressive symptoms (respiratory-, digestive-, great vessel-, and/or vocal fold-related). CXR and CT are the most appropriate and widely used diagnostic modalities. Thyroid scans are of limited utility, however, occasionally helpful [8].

Levothyroxine suppressive therapy fails to effectively achieve size reduction in the vast majority of instances. Whereas radioactive iodine not only fails in reducing the size of the goiter, but might also lead to potentially life-threatening airway obstruction caused by considerable swelling of the gland secondary to radioactive iodine-induced thyroiditis. Accordingly, Surgery remains the standalone therapeutic modality for RG [9]. Nevertheless, whether surgery should be performed on routine basis for RG regardless of symptoms remains an area of ongoing debate. Some consider the diagnosis of RG an indication for thyroidectomy as the risk of bleeding inside the goiter and/or the risk of malignant transformation are potentially serious consequences, and the morbidity from surgery is very low [10], [11], [12]. Others consider surgery for RG significantly more morbid than that for its cervical counterpart. Therefore, they only justify surgery in asymptomatic patients in the presence of suspicion for malignancy [13].

Despite the vast majority of RGs being successfully operated via a cervical approach, an extra-cervical access (manubrutomy, sternotomy, or thoracotomy) may be required in 2–5% of cases, even in the hands of an experienced surgeon [5].

Several factors have been found to considerably increase the likelihood of requiring an extra-cervical approach [14]. These include 1. The presence of an ectopic (primary intrathoracic) goiter 2. The presence of malignancy. This is related to the potential for extrathyroidal extension and the need to achieve oncological resection, or to perform a mediastinal nodal dissection. As well as avoiding malignant seeding 3. Extension of the goiter into the posterior mediastinum, and 4. Extension of the goiter below the aortic arch. Previous neck surgery and the frequent finding of adherences with surrounding tissues is another factor that potentially favors an extra-cervical approach [15], [16]. A heavy gland reflected by its CT volume is also an important determinant of the surgical approach [17].

To conclude, type II respiratory failure is an unprecedented manifestation of RG. A high index of suspicion for a recurrent symptomatic RG should be maintained in patients with a prolonged history of any procedure short of a total or near-total thyroidectomy. CT plays a key role in determining the likelihood of requiring an extra-cervical approach in RG. Regardless of CT findings, a cautious attempt to remove RG through a cervicotomy should always be made, on account of reduced risk of surgical and aesthetic harm. Furthermore, surgeon's experience is the most important denominator in thyroid surgery. If RG is operated by an experienced surgeon, familiar with its unique pitfalls, the assistance of a thoracic surgeon may only be required in a few cases.

## Provenance and peer review

Not commissioned, externally peer-reviewed.

## Source of funding

None.

## Ethical approval

The Hashemite University Institutional Review Board (IRB) approval was obtained.

## Consent

Written informed consent was obtained from the patient for publication of this case report and accompanying images. A copy of the written consent is available for review by the Editor-in-Chief of this journal on request.

## Guarantor

Dr. Sohail Bakkar accountable for all aspects of the work in ensuring that questions related to the accuracy or integrity of any part of the work are appropriately investigated and resolved.

## Registration of research studies

NA.

## CRediT authorship contribution statement

**SB**: study concept and design, data interpretation, paper writing, final approval and accountability for all aspects of the work. **QH and RH**: data collection, analysis, review of literature, final approval and accountability for all aspects of the work. **GD**, **TP**, **MT**: critical revision, paper writing, final approval and accountability for all aspects of the work.

## Declaration of competing interest

All authors have completed the disclosure form for IJS Case Reports, and have no conflicts of interest to declare.
